# Effects of Mixing Garlic Skin on Fermentation Quality, Microbial Community of High-Moisture *Pennisetum hydridum* Silage

**DOI:** 10.3389/fmicb.2021.770591

**Published:** 2021-11-08

**Authors:** Juncai Chen, Guohao Huang, Hanlin Xiong, Hao Qin, Haonan Zhang, Yawang Sun, Xianwen Dong, Yan Lei, Yongju Zhao, Zhongquan Zhao

**Affiliations:** ^1^College of Animal Science and Technology, Southwest University, Chongqing, China; ^2^Chongqing Key Laboratory of Herbivore Science, Chongqing, China; ^3^Chongqing Academy of Animal Science, Chongqing, China; ^4^Chengdu Agricultural College, Chengdu, China

**Keywords:** garlic skin, *Pennisetum hydridum*, high-moisture silage, silage quality, bacterial community

## Abstract

Garlic skin, a by-product of garlic processing, was supposed to improve the fermentation quality of high-moisture silages because of its low moisture content and active compounds. Thus, fermentation and microbial characteristics of high-moisture Pennisetum hydridum ensiled with the addition of 0, 10, 20, and 30 wt% garlic skin (on a fresh matter basis) were analyzed during a 60-days fermentation. Results showed that the addition of garlic skin increased the dry matter content and lactic acid production, and decreased the pH and ammonia-N content of the silage. Adding garlic skin changed the relative abundance of bacterial communities with an increase in Lactobacillus and a decrease in Clostridium relative abundance. In conclusion, co-ensiling of high-moisture Pennisetum hydridum with garlic skin could be a simple approach to improve the silage quality and nutrients preservation.

## Introduction

Garlic (*Allium sativum* L.) is a flavoring ingredient universally used with an annual production of approximately 20 million tons in the world (Lee et al., 2017). The by-products of garlic processing (garlic skin and straw) account for 25–30% of the weight of the raw material (Arzanlou and Bohlooli, 2010) but are mostly discarded or incinerated, which causes environmental pollution (Kallel et al., 2014). It was reported that garlic skin contains similar antimicrobial and antioxidative compounds as the garlic bulb, such as alliin (Louis et al., 2012), N-trans-coumaroyloctopamine and N-trans-feruloyloctopamine (Wu et al., 2015), and polyphenols (Kallel et al., 2014). There is increased interest in utilizing garlic skin in animal husbandry in recent years. Although garlic skin has a slightly pungent odor, Zhu et al. (2021) find that supplementation of 80 g/kg dry matter (DM) garlic skin did not influence the DM intake and improved the growth performance of lambs. However, garlic skin is not suitable to be fed alone owing to its relatively low nutritional values.

*Pennisetum hydridum* (*P. hydridum*), also known as king grass or *Hybrid Pennisetum*, is a perennial gramineous forage species, which has been widely planted in the tropics and subtropics of China. *P. hydridum* has high fresh biomass yields (85–300 t ha^–1^) and strong resistance to environmental threats (Tan et al., 2021) and is commonly used as a forage source for ruminants because of its high palatability and nutritional values (Li et al., 2014). Because of the seasonal imbalance availability of *P. hydridum*, ensiling is an ideal preservation approach that allows year-round quality forage supplements for ruminants. However, the moisture content of fresh *P. hydridum* can be higher than 80%, which enhances the risk of clostridial fermentation, extensive proteolysis, and nutrient loss during ensiling (Yan et al., 2019). Wilting prior to ensiling is commonly used to reduce the moisture of the fresh materials, but it may cause extra nutritional losses, and the effectiveness highly depends on weather conditions (Franco et al., 2017).

Mixing with a low-moisture material is demonstrated to be an efficient way to optimize the moisture content and improve the fermentation quality of high-moisture silages (Yan et al., 2019; He et al., 2020b). Besides the active compounds, garlic skin is characterized by low-moisture content. It is reasonable to hypothesize that addition of garlic skin could improve the fermentation quality of high-moisture *P. hydridum* silage by its active compounds and low moisture content and, thus, the inhibition of undesirable microbial growth during ensiling. To the best of our knowledge, no study has been conducted to estimate the effects of the addition of garlic skin on the silage fermentation quality. Therefore, the objectives of the current study were to investigate silage fermentation characteristics and bacterial community of high-moisture fresh *P. hydridum* when ensiled with the garlic skin at different ratios.

## Materials and Methods

### Raw Materials and Silage Preparation

The *P. hydridum* was manually harvested in July from an experimental field (29°49′N, 106°25′E) of Southwest University, Chongqing, China. The collected materials were chopped into 2–3 cm with a manual forage chopper without wilting. Garlic skin was from Tengda Farming Co., Ltd., (Chongqing, China). The chemical composition and microbial population of materials before ensiling are shown in Table 1. Silage treatments were (1) 100 wt% *P. hydridum* (GS0); (2) 90 wt% *P. hydridum*+ 10 wt% garlic skin (GS10); (3) 80 wt% *P. hydridum*+ 20 wt% garlic skin (GS20); and (4) 70 wt% *P. hydridum*+ 30 wt% garlic skin (GS30) on a fresh matter (FM) basis. After thorough mixing, each mixture (approximately 300 g) was packed into vacuum-sealed plastic film bags (dimensions 200 mm × 300 mm, Dongguan Bojia Packaging Co., Ltd., Dongguan, China). In total, 48 bags (4 treatments × 4 ensiling time points × 3 replicates) were prepared and stored at ambient temperature (25–35°C).

**TABLE 1:** Chemical composition and microbial population of *Pennisetum hydridum* and garlic skin before ensiling (±SD, *n* = 3).

Item		*Pennisetum hydridum*	Garlic skin
Chemical composition			
	Dry matter (% FM)	16.35 ± 1.76	84.78 ± 0.17
	Crude protein (% DM)	12.02 ± 0.06	4.06 ± 0.13
	NDF (% DM)	69.54 ± 2.13	52.23 ± 1.38
	ADF (% DM)	40.38 ± 1.36	42.79 ± 0.03
	Water soluble carbohydrate (% DM)	9.98 ± 0.55	9.08 ± 0.05
Microbial population			
	Lactic acid bacteria (Log_10_ CFU/g FM)	5.51 ± 0.19	5.60 ± 0.34
	Yeasts (Log_10_ CFU/g FM)	2.53 ± 0.13	3.52 ± 0.32
	Molds (Log_10_ CFU/g FM)	4.09 ± 0.01	3.69 ± 0.31
	Coliform bacteria (Log_10_ CFU/g FM)	6.32 ± 0.18	5.92 ± 0.59

*FM, fresh matter; DM, dry matter; NDF, neutral detergent fiber; ADF, acid detergent fiber; CFU, colony-forming unit.*

### Analysis of Chemical Composition and Fermentation

Pre- and post-ensiling materials were oven-dried at 65°C for 48 h to determine DM content and then ground (1 mm sieve) for chemical analysis. The DM and crude protein (CP) contents were determined and calculated according to the methods of the Association of Official Analytical Chemists (AOAC, 1990). The contents of acid detergent fiber (ADF) and neutral detergent fiber (NDF) were analyzed according to Van Soest et al. (1991) using an ANKOM A220 fiber analyzer. The water-soluble carbohydrate (WSC) content was determined by the anthrone method (Murphy, 1958).

A sample of approximately 20 g was taken from each bag and stored at 4°C for 24 h after mixing with 180 mL distilled water and then filtered through four layers of cheesecloth. The pH of the filtrate was measured using a pH meter (PHS-3E, INESA, Shanghai, China). The filtrates were centrifuged for 10 min at 10,000 × g, and the supernatants were filtered with a membrane (0.22 μm). The lactic, acetic, propionic, and butyric acids were determined using high-performance liquid chromatography (KC-811 column, Shodex; mobile phase, 3 mM perchloric acid; flow rate, 1.0 mL/min; temperature: 50°C) (Li et al., 2019). The ammonia-N (NH_3_-N) concentration was determined using the phenol-hypochlorite reaction method (Broderick and Kang, 1980) and expressed as per the total nitrogen (g/kg total nitrogen).

### Microbial Population Analysis

The microbial population was analyzed by using the plate count method as described by Wang Y. et al. (2019). Briefly, the sample of 20 g was taken from each bag and blended with 180 mL sterilized saline solution (8.5 g/L NaCl) for around 30 min and serially diluted from 10^–1^ to 10^–7^ in sterile saline solution. The population of lactic acid bacteria and coliform bacteria were separately incubated on Man Rogosa Sharpe agar and Violet Red Bile agar, and yeasts and molds were separately incubated on Rose Bengal agar. Colonies were counted as numbers of viable microorganisms in the colony-forming unit (CFU) per gram of FM.

### DNA Extraction, PCR Amplification, and Sequencing Analysis

Total DNA was extracted from the silage samples using the HiPure Soil DNA Kits (Magen, Guangzhou, China) according to the manufacturer’s instructions. The PCR reactions were conducted in triplicate 50 μL mixture (10 μL of 5 × Q5@ reaction buffer, 10 μL of 5 × Q5@ high GC enhancer, 1.5 μL of 2.5 mM dNTPs, 1.5 μL of each primer, 0.2 μL of Q5@ high-fidelity DNA polymerase, and 50 ng of template DNA). The V3-V4 regions of 16S rDNA were amplified using primers 341F (CCTACGGGNGGCWGCAG) and 806R (GGACTACHVGGGTATCTAAT) by PCR (95°C for 5 min, followed by 30 cycles at 95°C for 1 min, 60°C for 1 min, and 72°C for 1 min and a final extension at 72°C for 7 min) (Dong et al., 2020). Related PCR reagents were from New England Biolabs, United States. The amplified PCR products were extracted from 2% agarose gels and purified using the AxyPrep DNA Gel Extraction Kit (Axygen Biosciences, Union City, CA, United States) according to the manufacturer’s instructions and followed by paired-end sequencing (PE250) on an Illumina platform (Illumina, United States) according to the standard protocols. The sequences data reported in this study were archived in the Sequence Read Archive (SRA) with the BioProject accession number PRJNA755145.

### Bioinformatics Analysis

The original sequencings were quality filtered by removing the sequence containing more than 10% of unknown nucleotides and the sequence containing more than 50% of bases with *Q*-value > 20 by FASTP (version 0.18.0) and merged by FLASH (version 1.2.11), respectively. The paired and clean reads were merged as raw tags using FLASH with a minimum overlap of 10 bp and mismatch error rates of 2% as stated in Wang C. et al. (2019). The raw tags were filtered using the QIIME (version 1.9.1) pipeline and chimeric sequences were removed using the UCHIME algorithm. The clean tags were clustered into operational taxonomic units (OTUs) of ≥ 97% similarity using the UPARSE (version 9.2.64) pipeline. The taxonomy of each representative OTU sequence was analyzed by Ribosome Database Project (RDP) classifier (version 2.2) based on the SILVA database (version 132). The alpha diversity indexes, including Chao1, ACE, Shannon, Simpson, and Good’s coverage, were calculated in QIIME (version 1.9.1). Principal coordinate analysis of unweighted UniFrac distances was performed and plotted in R. The Kyoto Encyclopedia of Genes and Genomes (KEGG) pathway analysis of OTUs was performed using Tax4Fun for the prediction of the metabolic function of microorganisms (Wang C. et al., 2019).

### Statistical Analysis

Microbial populations of forage and silage were log-transformed prior to statistical analysis. The effects of mixing *P. hydridum* with garlic skin on chemical composition, fermentation, microbial population, the dominant genus of bacteria, and the predicted bacterial functions were evaluated by one-way ANOVA with Tukey’s HSD *post-hoc* tests in the R program (v. 3.1.1).^1^ Statistical significance of the principal coordinate analysis was performed using analysis of similarities (ANOSIM) and permutational multivariate analysis of variance (ADONIS) in R program. A *P*-value of less than 0.05 was considered to be a significant difference.

## Results

### Chemical Composition, Fermentation Characteristics, and Microbial Population During Ensiling

The dynamic changes of the chemical composition of *P. hydridum* and garlic skin silage during the ensiling process are shown in Table 2. The silage DM content was increased (*P* < 0.01), and the CP content was decreased (*P* < 0.01) with the increasing garlic skin proportion. The CP contents in all silage treatments remained relatively stable during the 60 days of ensiling in the current study. The NDF contents in *P. hydridum* silage mixed with garlic skin decreased (*P* < 0.01) compared with the *P. hydridum* silage ensiled alone, and the ADF contents were not influenced by the addition of garlic skin.

**TABLE 2:** Chemical composition of *Pennisetum hydridum* ensiled with garlic skin.

Item		Treatments^1^				SEM	*P*-value
		GS0	GS10	GS20	GS30		
Ensiled for 7 days							
	DM (% FM)	17.38^d^	23.25^c^	30.26^b^	36.47^a^	0.67	<0.01
	Crude protein (% DM)	12.37^a^	8.92^b^	7.07^bc^	5.77^c^	0.71	<0.01
	NDF (% DM)	66.52^a^	63.60^a^	59.69^b^	56.07^c^	1.01	<0.01
	ADF (% DM)	39.39	40.00	40.62	40.58	1.48	0.92
Ensiled for 14 days							
	DM (% FM)	16.15^d^	22.22^c^	29.56^b^	35.26^a^	1.07	<0.01
	Crude protein (% DM)	10.89^a^	8.10^b^	6.51^c^	5.49^c^	0.42	<0.01
	NDF (% DM)	66.34^a^	62.02^ab^	57.03^ab^	53.86^b^	3.02	0.07
	ADF (% DM)	38.63	39.63	39.72	39.49	1.19	0.91
Ensiled for 30 days							
	DM (% FM)	15.09^d^	21.74^c^	29.00^b^	34.66^a^	0.42	<0.01
	Crude protein (% DM)	11.83^a^	8.96^b^	7.33^c^	5.77^d^	0.26	<0.01
	NDF (% DM)	65.34^a^	61.79^b^	56.46^c^	52.22^d^	1.08	0.40
	ADF (% DM)	39.28	38.41	38.59	38.81	1.47	0.98
Ensiled for 60 days							
	DM (% FM)	14.85^d^	20.67^c^	28.95^b^	34.24^a^	0.80	<0.01
	Crude protein (% DM)	12.41^a^	9.21^b^	7.89^c^	6.61^d^	0.37	<0.01
	NDF (% DM)	62.95^a^	61.27^a^	55.78^b^	52.26^c^	1.04	0.12
	ADF (% DM)	38.66	37.30	37.40	38.31	1.74	0.93

*Different letters following numbers indicate a significant difference (P < 0.05). FM, fresh matter; DM, dry matter; NDF, neutral detergent fiber; ADF, acid detergent fiber; SEM, standard error of means.*

*^1^GS0, 100 wt% Pennisetum hydridum; GS10, 90 wt% Pennisetum hydridum + 10 wt% garlic skin; GS20, 80 wt% Pennisetum hydridum + 20 wt% garlic skin; GS30, 70 wt% Pennisetum hydridum +30 wt% garlic skin.*

**FIGURE 1:**
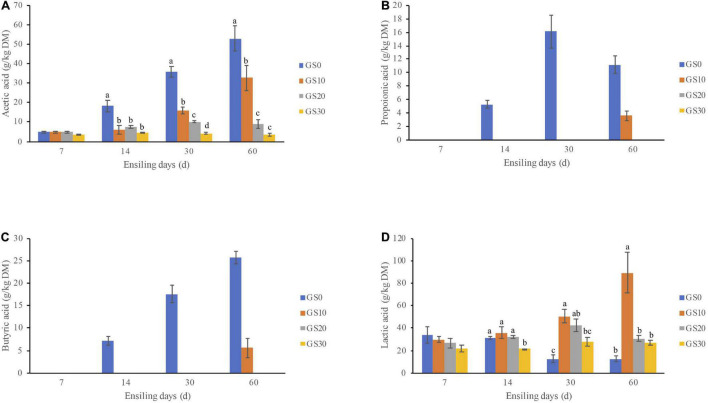
Dynamic changes of organic acids, including acetic acid **(A)**, propionic acid **(B)**, butyric acid **(C)**, and lactic acid **(D)** of *Pennisetum hydridum* ensiled with garlic skin (GS0, 100 wt% *Pennisetum hydridum*; GS10, 90 wt% *Pennisetum hydridum*+ 10 wt% garlic skin; GS20, 80 wt% *Pennisetum hydridum*+ 20 wt% garlic skin; GS30, 70 wt% *Pennisetum hydridum*+30 wt% garlic skin). Different letters indicate a significant difference (*P* < 0.05).

The dynamics of the fermentation profiles and microbial population of *P. hydridum* and garlic skin silage are shown in Table 3, and the dynamics of organic acids are shown in Figure 1. Overall, the addition of garlic skin significantly influenced the pH, fermentation products, and microbial population of silages. The pH values declined in all silage treatments with the ensiling progressed, and the final pH value of the GS0 silage (5.08) after 60 days fermentation was greater compared with GS20 (4.08) and GS30 silages (4.13, *P* < 0.01) with an intermediate value for GS10 silage (4.39). The lactic acid contents of GS0 silage decreased from days 7 to 60 of ensiling, and that of *P. hydridum* silage mixed with garlic skin increased days 7–30 of ensiling. The GS10 silage had the greatest lactic acid contents at days 60 of ensiling (*P* < 0.01). The acetic acid and NH_3_-N contents were greatest in GS0 silage (*P* < 0.01) at all-time points, and the contents of propionic acid and butyric acid were not detectable in GS10, GS20, and GS30 silages. In general, the lactic acid bacteria counts decreased as the ensiling progressed, and that was greatest in GS10 silage (*P* < 0.01), followed by GS10 and GS20 and least in GS0 silage at days 60 of ensiling. The counts of yeasts and molds were below the detectable levels after days 14 of ensiling. Although the counts of coliform bacteria declined consistently, it was still greater than 3 log_10_ cfu/g of FM in all silage treatments after the 60 days ensiling.

**TABLE 3:** pH, NH_3_-N and microbial population of *Pennisetum hydridum* ensiled with garlic skin.

Item		Treatments^1^				SEM	*P*-value
		GS0	GS10	GS20	GS30		
**Ensiled for 7 days**							
	pH	6.51^a^	5.40^b^	5.36^b^	5.12^b^	0.14	<0.01
	NH_3_-N (g/kg TN)	167.15^a^	90.63^b^	89.00^b^	92.92^b^	2.19	<0.01
	Lactic acid bacteria (Log_10_ CFU/g FM)	7.40^a^	7.10^ab^	7.27^b^	7.03^b^	0.07	0.03
	Yeasts (Log_10_ CFU/g FM)	3.68	3.48	3.667	3.89	0.17	0.48
	Molds (Log_10_ CFU/g FM)	<2	2.68^b^	2.96^ab^	3.66^a^	0.22	0.04
	Coliform bacteria (Log_10_ CFU/g FM)	6.66^a^	6.24^b^	6.04^b^	5.99^b^	0.11	0.01
**Ensiled for 14 days**							
	pH	6.32^a^	5.05^b^	4.85^b^	4.87^b^	0.14	<0.01
	NH_3_-N (g/kg TN)	289.51^a^	143.54^b^	130.19^b^	110.59^b^	4.02	<0.01
	Lactic acid bacteria (Log_10_ CFU/g FM)	7.12^b^	7.32^ab^	7.25^b^	7.52^a^	0.07	0.03
	Yeasts (Log_10_ CFU/g FM)	<2	<2	<2	<2	−	−
	Molds (Log_10_ CFU/g FM)	<2	<2	<2	<2	−	−
	Coliform bacteria (Log_10_ CFU/g FM)	5.61^a^	5.21^b^	5.30^b^	5.50^a^	0.06	<0.01
	**Ensiled for 30 days**						
	pH	5.51^a^	4.42^b^	4.19^c^	4.27^c^	0.04	<0.01
	NH_3_-N (g/kg TN)	351.66^a^	163.04^b^	118.62^bc^	91.96^c^	2.28	<0.01
	Lactic acid bacteria (Log_10_ CFU/g FM)	6.29^c^	7.27^a^	6.99^b^	6.95^b^	0.07	<0.01
	Yeasts (Log_10_ CFU/g FM)	<2	<2	<2	<2	−	−
	Molds (Log_10_ CFU/g FM)	<2	<2	<2	<2	−	−
	Coliform bacteria (Log_10_ CFU/g FM)	5.45^a^	5.16^b^	5.06^b^	5.43^a^	0.06	<0.01
**Ensiled for 60 days**							
	pH	5.08^a^	4.39^b^	4.08^c^	4.13^c^	0.08	<0.01
	NH_3_-N (g/kg TN)	406.94^a^	188.81^b^	110.64^c^	85.68^c^	3.20	<0.01
	Lactic acid bacteria (Log_10_ CFU/g FM)	5.68^c^	7.05^a^	6.59^b^	6.29^b^	0.12	<0.01
	Yeasts (Log_10_ CFU/g FM)	<2	<2	<2	<2	−	−
	Molds (Log_10_ CFU/g FM)	<2	<2	<2	<2	−	−
	Coliform bacteria (Log_10_ CFU/g FM)	3.94	4.14	3.95	3.88	0.17	0.75

*Different letters following numbers indicate a significant difference (P < 0.05). FM, fresh matter; CFU, colony-forming unit; TN, total nitrogen; SEM, standard error of means.*

*^1^GS0, 100 wt% Pennisetum hydridum; GS10, 90 wt% Pennisetum hydridum + 10 wt% garlic skin; GS20, 80 wt% Pennisetum hydridum + 20 wt% garlic skin; GS30, 70 wt% Pennisetum hydridum +30 wt% garlic skin.*

### Bacterial Diversity and Abundance During Ensiling

**FIGURE 2:**
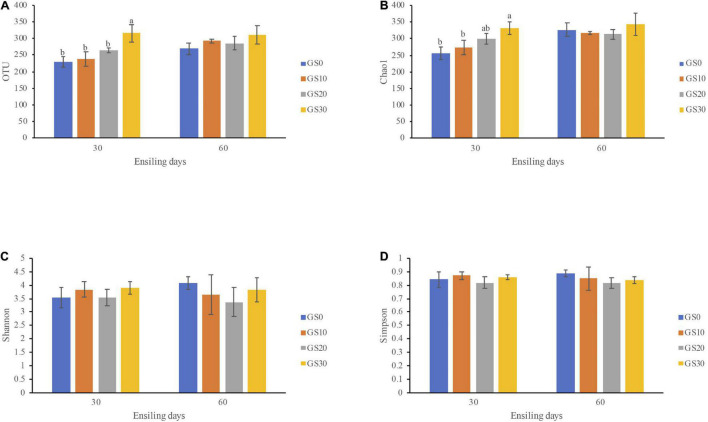
Alpha diversity indices of bacterial community including OTU **(A)**, Chao1 **(B)**, Shannon **(C)**, and Simpson **(D)** of *Pennisetum hydridum* ensiled with garlic skin (GS0, 100 wt% *Pennisetum hydridum*; GS10, 90 wt% *Pennisetum hydridum*+ 10 wt% garlic skin; GS20, 80 wt% *Pennisetum hydridum*+ 20 wt% garlic skin; GS30, 70 wt% *Pennisetum hydridum*+30 wt% garlic skin). Different letters indicate a significant difference (*P* < 0.05).

**FIGURE 3:**
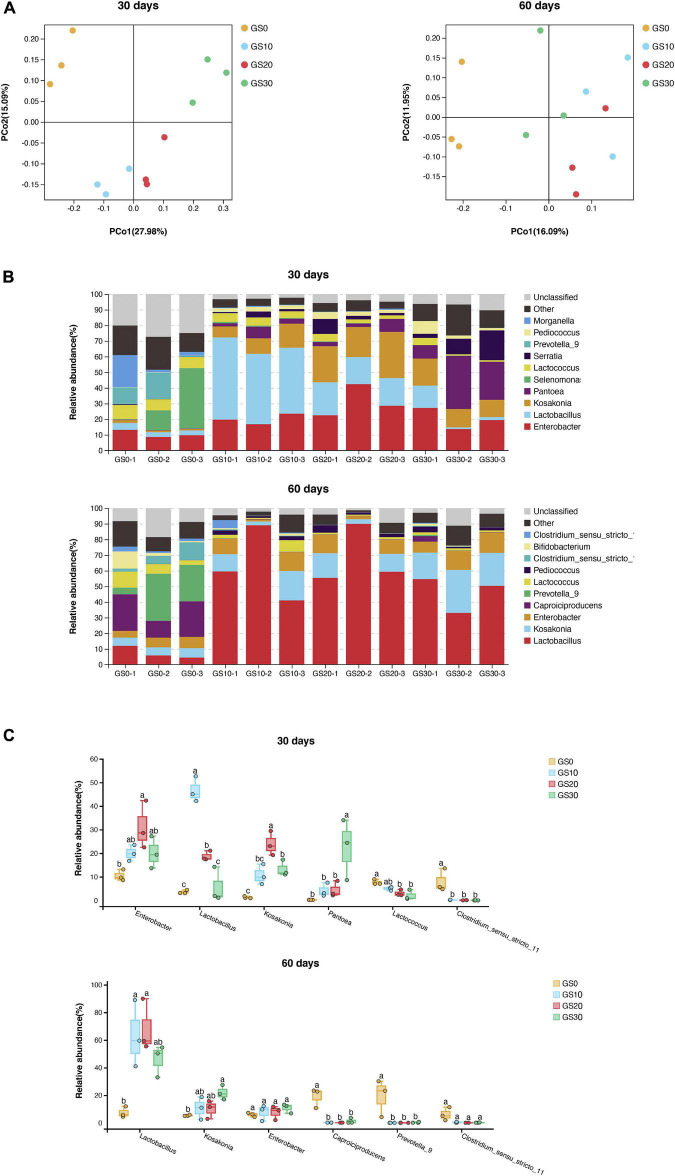
Principal coordinate analysis **(A)**, relative abundance by genus **(B)**, and dominant genus (**C**, > 5% relative abundance at least one group) of bacterial community for *Pennisetum hydridum* ensiled with garlic skin at days 30 and 60 of ensiling (GS0, 100 wt% *Pennisetum hydridum*; GS10, 90 wt% *Pennisetum hydridum*+ 10 wt% garlic skin; GS20, 80 wt% *Pennisetum hydridum*+ 20 wt% garlic skin; GS30, 70 wt% *Pennisetum hydridum*+30 wt% garlic skin). Different letters indicate a significant difference (*P* < 0.05).

The alpha diversity of the bacterial community of *P. hydridum* ensiled with garlic skin is shown in Figure 2. All Good’s coverage estimates of silages were around 0.99, indicating most of the bacteria were detected. The bacterial community richness indices, OTU, and Chao1 increased in all treatment silages from days 30 to 60 of ensiling, among which GS30 silage had the greatest microbial richness. Moreover, the bacterial diversity of GS10, GS20, and GS30 silages declined while that of GS0 silage rose from days 30 to 60 of ensiling as indicated by the Shannon and Simpson indices. The principal coordinate analysis based on the unweighted UniFrac distances was carried out to estimate the variance of the bacterial community, which revealed that the GS0 silage was significantly separated from GS10, GS20, and GS30 at both days 30 and 60 of ensiling (*P* < 0.01, Figure 3A). The relative abundance of bacterial communities at the genus level and dominant genus (>5% relative abundance at least one group) are shown in Figures 3B,C, respectively. Overall, the addition of garlic skin considerably changed the relative abundance of bacterial communities compared with *P. hydridum* silage ensiled alone. At days 30 of ensiling, the *Enterobacter* (20.0–31.1%), *Lactobacillus* (5.7–46.6%), and *Kosakonia* (13.3–23.9%) were the dominant microbes in GS10, GS20, and GS30 silages while GS0 silage was dominated by *Selenomonas* (17.3%) and *Enterobacter* (10.5%). However, the relative abundance of *Enterobacter* at days 60 of ensiling decreased to 7.6–10.8% and the abundance of *Lactobacillus* increased to 45.8–68.2% in GS10, GS20, and GS30 silages.

The relative abundance of *Clostridium* was greatest in GS0 silages, and garlic skin significantly increased the relative abundance of *Kosakonia*, *Pantoea*, *Enterobacter*, and *Lactobacillus* at days 30 of ensiling (*P* < 0.01). At days 60 of ensiling, the relative abundance of *Lactobacillus* and *Kosakonia* were still lower in GS0 silage compared with *P. hydridum* ensiled with garlic skin. Furthermore, the relative abundance of *Prevotella* and *Caproiciproducens* in GS0 silage was significantly greater than other silage treatments (*P* < 0.01).

### Predicted Potential Functions

**FIGURE 4:**
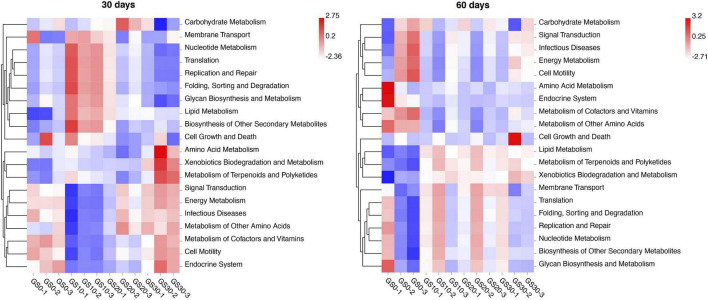
Bacterial functional predictions (top 20 in abundance) for *Pennisetum hydridum* ensiled with garlic skin at days 30 and 60 of ensiling obtained with Tax4Fun (GS0, 100 wt% *Pennisetum hydridum*; GS10, 90 wt% *Pennisetum hydridum*+ 10 wt% garlic skin; GS20, 80 wt% *Pennisetum hydridum*+ 20 wt% garlic skin; GS30, 70 wt% *Pennisetum hydridum*+30 wt% garlic skin).

To gain knowledge of the potential metabolic pathways involved in the garlic skin and *P. hydridum* silage ensiling process, bacterial function prediction analysis was performed by Tax4Fun based on the 16S rRNA gene sequences. At the first level of the KEGG pathway, the most abundance pathways were metabolism (∼79%) and genetic information processing (∼11%), followed by cellular processes (∼5%), environmental information processing (∼4%), human diseases (<1%), and organismal systems (<1%), which were not highly varied among silage treatments or between days 30 and 60 of ensiling. At the second level of the KEGG pathway, the relative abundance pathways of signal transduction, metabolism of cofactors and vitamins, energy metabolism, and infectious diseases were lower, and the relative abundance pathways of nucleotide metabolism, translation, and replication and repair were greater in GS10 silage compared with other silages at d 30 of ensiling (Figure 4). At days 60 of ensiling, the relative abundance pathways of lipid metabolism were lower, and the relative abundance pathways of metabolism of cofactors and vitamins were greater in GS0 silage compared with other silages.

## Discussion

DM content is a key factor affecting the silage fermentation quality. The inappropriate DM content of the ensiling silage results in seepage and an undesirable fermentation (DM < 28%) or difficulty to pack and poorly fermented (DM > 40%) (Guyader et al., 2018). The DM content of *P. hydridum* in the current study (16.35%) is similar to that reported in a previous study (Li et al., 2014) and is far lower than the ideal DM content for silage processing, implying the difficulty to obtain high-quality silage without further treatment. Thus, mixing high-moisture *P. hydridum* with dry materials, e.g., garlic skin, could be an efficient strategy to reduce the moisture content and improve the fermentation quality. The CP content of *P. hydridum* is higher than that of ryegrass (10.74% DM) and dried corn stover (6.08% DM, Yan et al., 2019), suggesting that *P. hydridum* is good forage for ruminants. The relatively low CP content of garlic skin (4.06 % DM) suggests that the high mixing ratio of garlic skin might reduce the nutritional value of the silage. The *P. hydridum* has stemmy structures and accordingly has relatively high fiber content, which is in line with the previous study (Shah et al., 2017). Additionally, the epiphytic microbial community and WSC content are two crucial factors that affect the fermentation quality. For well-preserved silage, epiphytic lactic acid bacteria should reach counts of at least 10^5^ cfu/g of FM (Cai et al., 1999), and WSC content should be greater than 5% DM for propagation and growth of lactic acid bacteria during ensiling (Smith, 1962). In the current study, although both *P. hydridum* and garlic skin had sufficient WSC concentration and epiphytic lactic acid bacteria, medium counts of undesirable microorganisms, e.g., yeasts, molds, and coliform bacteria, were distributed in both forages, which implies the risk of poor fermentation.

As expected, the differences of DM and CP content among silage treatments were highly related to the DM and CP content of *P. hydridum* and garlic skin in the current study. In addition, DM contents in all silages consistently decreased as the ensiling progressed, which is in accordance with previous studies (Li et al., 2018; Dong et al., 2020). The DM reduction primarily occurs at the early stage of ensiling due to the microbial breakdown of substrates into liquid and gases, which would be gradually inhibited as the ensiling progressed (Desta et al., 2016). Nevertheless, CP contents remained relatively stable during the 60 days of ensiling, which is consistent with the results reported in a previous study (He et al., 2020b). The CP content is calculated based on the total nitrogen of the silage and the proteolysis during ensiling mainly changes the protein to non-protein nitrogen compounds instead of total nitrogen (Fijałkowska et al., 2015), which may partly explain the relatively stable CP content in the current study. Similar to the results reported by Li et al. (2018), the NDF and ADF contents decreased over a 60-days fermentation. A previous study shows that the addition of cellulase could reduce the content of NDF and ADF in *P. hydridum* silages (Li et al., 2014). In the future, it would be interesting to evaluate if the addition of garlic skin combined with cellulose could further decrease the NDF and ADF content of the silage.

The pH value is a simple but important criterion to evaluate the silage fermentation quality. pH ≤ 4.2 is generally regarded as an indicator for well-persevered silages (McDonald et al., 1991). High-moisture silage (>70%) and moderate pH (>4.5) favor clostridial fermentation (Muck, 1988). In line with that, the poor fermentation quality of high-moisture *P. hydridum* silage (GS0) was observed in the current study. The final pH value of the GS0 silage (5.08) after 60 days fermentation was still greater than the threshold pH value despite the consistent decline of pH during the fermentation process. The accumulation of acetic, propionic, and butyric acid content and decreased lactic acid in GS0 silage indicate the failure to inhibit the growth of spoilage organisms, such as clostridia that could ferment lactic acid to butyric acid (He et al., 2020b). Generally, the butyric acid content of silage < 10 g/kg DM is considered desirable, and excessive butyric acid in silage can be associated with reduced silage intake and increased risk of clinical ketosis (Driehuis et al., 2018). The butyric acid of GS0 silage, however, was greater than 20 g/kg DM after 60-days fermentation. During ensiling, the protein is degraded to free amino acids, amines, and ammonia by the proteolytic plant enzymes in the early phase and by bacteria when the pH drops below 5.0, which decreases the efficiency of nitrogen utilization for ruminants (Fijałkowska et al., 2015). Thus, the highest NH_3_-N content of GS0 silage among groups reflects the greatest proteolysis during ensiling. Collectively, these results indicate the GS0 silage is poorly fermented and not suitable to be used as ruminant feed.

In the current study, the addition of garlic skin significantly improved the fermentation quality of silage as indicated by lower pH value, acetic acids, and NH_3_-N contents, which is similar to the effects of silage additives (e.g., sucrose, glucose, molasses, or glucose) on *P. hydridum* silage fermentation quality (Li et al., 2014). Moreover, propionic and butyric acid were not detectable in the *P. hydridum* silages mixed with garlic skin throughout the ensiling process. Clearly, these results indicate that garlic skin inhibited the growth of clostridia and changed the fermentation type. Of note, the DM content of GS10 silage was still considerably lower than the appropriate DM content for the good fermentation quality, but the fermentation quality has been significantly improved. Besides the low moisture content, the garlic skin may exert beneficial effects *via* its active constituents, e.g., phenylpropanoid (Ichikawa et al., 2003) or alliin (Louis et al., 2012), which have been reported to possess antibacterial activity (Choi et al., 2007; Hemaiswarya and Doble, 2010). Interestingly, the lactic acid contents of GS10 were greater but the pH value was higher than that of those of GS20 and GS30 after 60 days of ensiling. The pH of silage is most correlated with the lactic acid content and the buffering capacity of the materials (Kung et al., 2018), and *P. hydridum* has a relatively high buffering capacity (Yahaya et al., 2004), which could be the reason for the higher pH value of GS10 silage. In the current study, the counts of yeasts and molds were 2.53–4.09 log_10_ cfu/g FM in both fresh materials, but the population was decreased to below the detection level (<2 log_10_ cfu/g FM) after 14 days of ensiling in the current study. Coliform bacteria are the major competitor of lactic acid bacteria and are responsible for silage nutrient loss (Rauramaa et al., 1987). The counts of coliform bacteria were still high after 60 days of ensiling in all silages (3.88–4.11 log_10_ cfu/g FM) though the counts gradually declined with the decrease of pH during ensiling. It is suggested that coliform bacteria cannot be completely inhibited if the pH > 4.0 (Ni et al., 2015), and thus, the pH of the silages in the current study may not be low enough to inhibit its growth. To the best of our knowledge, the current study is the first to investigate the effects of garlic skin on silage fermentation quality. The findings not only provide a simple way to improve the fermentation quality of high-moisture silage without wilting or additive addition, but also open new avenues for the usage of garlic skin, which is mainly treated as industrial waste. Moreover, the strong aromatic odor of the well-fermented silage covered the pungent odor of garlic skin, which could potentially increase its palatability.

Basically, ensiling is a complex and dynamic microbial fermentation process. Ren et al. (2019) observed that the Chao index increased in the sugarcane top silage from days 2 to 60 and then decreased after days 60, which is similar to the findings in the current study. Moreover, the bacterial diversity declined from days 30 to 60 of ensiling in the *P. hydridum* silage mixed with garlic skin, but not in the *P. hydridum* silage ensiled alone. The high bacterial diversity of fresh material is gradually decreased with the lactic acid bacteria becoming predominant during successful silage fermentation (McDonald et al., 1991), which could be the reason for the reduction of bacterial diversity in *P. hydridum* silage mixed with garlic skin. These results show an increase in anaerobic stability in GS10, GS20, and GS30 silages but not in GS0 silage, which is in line with the fermentation quality data as mentioned above. Additionally, the principal coordinate analysis revealed that the GS0 silage was significantly separated from GS10, GS20, and GS30 at both days 30 and 60 of ensiling. Therefore, the improved fermentation quality in silages mixed with garlic skin seems to be related to the shift of the microbial community from undesirable microorganisms to favorable microorganisms.

The addition of garlic skin considerably changed the relative abundance of bacterial communities compared with *P. hydridum* silage ensiled alone. *Enterobacter* is a common genus during silage fermentation and is undesirable due to its competition for WSC and NH_3_-N production (Muck, 2010). As discussed above, the high abundance of *Enterobacter* at days 30 of ensiling could be related to the high moisture content of *P. hydridum* and medium pH value in the current study. He et al. (2020b) and Wu et al. (2020) also observed a high relative abundance of *Enterobacter* in *Pennisetum purpureum* silage (4.68–51.33%) and in high-moisture corn stalk silage (28.9–39.3%), respectively. However, the relative abundance of *Enterobacter* at days 60 of ensiling decreased, which could be associated with the decreased pH value caused by the significantly increased relative abundance of *Lactobacillus* (45.8–68.2%). Ensiling is an anaerobic process, and the higher relative abundance of anaerobes, e.g., *Lactobacillus*, produces lactic acid to inhibit the acid-intolerant *Enterobacter* (Dunière et al., 2013). Therefore, the addition of garlic skin in *P. hydridum* silage shifted the mixture of homo- and hetero-lactic fermentation to a more homo-lactic fermentation at days 60 of ensiling, which is more efficient in lactic acid production and results in fewer nutrients lost.

In the current study, the relative abundance of *Clostridium* was greatest in GS0 silages, which led to considerable nutrient losses and proteolysis and coincided with the highest butyric acid and NH_3_-N content in GS0 silages as aforementioned. He et al. (2020b) also observed that *Clostridium* was the first dominant genus in the high-moisture stylo silage. In addition, garlic skin increased the relative abundance of *Kosakonia* and *Pantoea* at days 30 of ensiling, and the relative abundance of *Kosakonia* was not substantially inhibited by the decreased pH value at days 60 of ensiling. *Kosakonia* is a novel reclassified branch from *Enterobacter* and is related to the fixation of nitrogen and production of xylanase (Chin et al., 2017). *Pantoea* has been reported to have negative correlations with silage pH, NH_3_-N concentration, and yeast and mold counts (Ogunade et al., 2018). He et al. (2020a) propose that *Kosakonia* and *Pantoea* might contribute to the reduction of NH_3_-N of silage. Furthermore, at days 60 of ensiling, the relative abundances of *Prevotella* and *Caproiciproducens* in GS0 silage were significantly greater than other silages. *Prevotella* is a dominant proteolytic bacterium in the rumen and could produce propionate during fermentation (Chen et al., 2019). *Caproiciproducens* is a recently defined genus and is also observed in Italian ryegrass and corn stover mixed silage (Yan et al., 2019) and stylo silage (Wang C. et al., 2019). The greater content of NH_3_-N, acetate, and butyrate could be related to the *Prevotella* and *Caproiciproducens* in GS0 silage, but the exact biological function of these bacteria in silage fermentation should be further investigated in future studies.

The bacterial functions prediction analysis showed that the several relative abundance pathways (i.e., signal transduction, energy metabolism, nucleotide metabolism, etc.) were significantly different in GS10 silage compared with other silages at days 30 of ensiling. These differences might be related to the greater growth of *Lactobacillus* in GS10 silage at days 30 of ensiling. Bai et al. (2021) also observed the opposite trend in nucleotide and energy metabolism in alfalfa silage, but the reason for this phenomenon is not clear and needs further investigation. At days 60 of ensiling, GS0 silage had lower relative abundance pathways of lipid metabolism and greater relative abundance pathways of metabolism of cofactors and vitamins compared with other silage treatments. Liu et al. (2020) suggest that the decreased *Firmicutes/Bacteroidetes* ratio may inhibit lipid metabolism–related gene expression. The significantly lower relative abundance of *Lactobacillus*, which is a classical type of *Firmicutes*, in GS0 silage could explain the lower lipid metabolism in the current study. Notably, the data about the predicted bacterial function should be interpreted cautiously, and direct mRNA measurements or transcriptomics should be employed to verify these metabolic pathways involving in silage fermentation (Keshri et al., 2018).

## Conclusion

The current study illustrates that co-ensiling of high-moisture *P. hydridum* with garlic skin could significantly decrease pH value, butyric acid, NH_3_-N, and increase lactic acid production. Moreover, the relative abundances of favorable bacteria, such as *Lactobacillus*, were increased while that of undesirable bacteria, such as *Clostridium* and *Enterobacter*, were decreased. These results suggest that the addition of garlic skin could be a simple way to improve the fermentation quality and preserve protein of high-moisture *P. hydridum* silage *via* optimizing the moisture content and, thus, the inhibition of undesirable microbial growth during ensiling. Future research focusing on the utilization in animal husbandry and the related economic analysis are needed for better application in practice, and metagenomic and metabolomic analysis should also be considered to better characterize bacterial species and their functionality during ensiling.

## Data Availability Statement

The datasets presented in this study can be found in online repositories. The names of the repository/repositories and accession number(s) can be found below: https://www.ncbi.nlm.nih.gov/, PRJNA755145.

## Author Contributions

JC, GH, and ZZ designed the study and wrote the manuscript. HX, HQ, HZ, and YS performed the experiments. YL analyzed the data. YZ was involved in the manuscript revision. All authors contributed to the article and approved the submitted version.

## Conflict of Interest

The authors declare that the research was conducted in the absence of any commercial or financial relationships that could be construed as a potential conflict of interest.

## Publisher’s Note

All claims expressed in this article are solely those of the authors and do not necessarily represent those of their affiliated organizations, or those of the publisher, the editors and the reviewers. Any product that may be evaluated in this article, or claim that may be made by its manufacturer, is not guaranteed or endorsed by the publisher.

## Footnotes

1
https://www.R-project.org/
